# Supplementary material for: Tracking cropland transitions: A comparative analysis of U.S. land cover change data
Source: PLoS One. 2025 Mar 18;20(3):e0313880. doi: 10.1371/journal.pone.0313880 (PMC11918356; doi:10.1371/journal.pone.0313880)
Supplement: S1 Table — Note that these definitions apply to the underlying land cover maps used to produce land change estimates. (DOCX) [file pone.0313880.s001.docx]

S1 Table. Land cover class definitions by dataset.

| **Dataset** | **Term** | **Definition** |
| --- | --- | --- |
| LCMAP | Cropland | Land in either a vegetated or unvegetated state used in production of food, fiber, and fuels. This includes cultivated and uncultivated croplands, hay lands, orchards, vineyards, and confined livestock operations. Forest plantations are considered as forests or woodlands (Tree Cover class) regardless of the use of the wood products. |
| LCMAP | Grass/Shrub | Land predominantly covered with shrubs and perennial or annual natural and domesticated grasses (e.g., pasture), forbs, or other forms of herbaceous vegetation. The grass and shrub cover must comprise at least 10% of the area and tree cover is less than 10% of the area. |
| NRI | Cropland | A land cover/use category that includes areas used for the production of adapted crops for harvest. Two subcategories of cropland are recognized: cultivated and non-cultivated. |
| NRI | Cultivated cropland | Cultivated cropland comprises land in row crops or close-grown crops and also other cultivated cropland, for example, hayland or pastureland that is in a rotation with row or close-grown crops. |
| NRI | Non-cultivated cropland | Non-cultivated cropland includes permanent hayland and horticultural cropland. |
| NRI | Hayland | A subcategory of cropland managed for the production of forage crops that are machine harvested. The crop may be grasses, legumes, or a combination of both. Hayland also includes land in set-aside or other short-term agricultural programs. |
| NRI | Horticultural cropland | A subcategory of cropland used for growing fruit, nut, berry, vineyard, and other bush fruit and similar crops. Nurseries and other ornamental plantings are included. |
| NRI | Pastureland | A land cover/use category of land managed primarily for the production of introduced forage plants for livestock grazing. Pastureland cover may consist of a single species in a pure stand, a grass mixture, or a grass-legume mixture... ...For the NRI, includes land that has a vegetative cover of grasses, legumes, and/or forbs, regardless of whether or not it is being grazed by livestock. |
| NRI | Rangeland | A land cover/use category on which the climax or potential plant cover is composed principally of native grasses, grasslike plants, forbs or shrubs suitable for grazing and browsing, and introduced forage species that are managed like rangeland. |
| Lark *et al*. 2020 | Grass/Pasture | Combines the following historical CDL categories: Pasture/Grass (code 62), Grassland Herbaceous (code 171), and Pasture/Hay (code 181). |
| Lark *et al*. 2020 | Cropland | Cropland was broadly defined as any area planted to cultivated row, closely grown, or horticultural crops and included cultivated fallow and alfalfa. |
| Potapov *et al*. 2022 | Cropland | Land used for annual and perennial herbaceous crops for human consumption, forage (including hay), and biofuel. This definition excludes perennial woody crops, permanent pastures, and shifting cultivation; additionally, the fallow length is limited to 4 years. This definition is largely consistent with the arable land category reported by the FAO. |

Note that these definitions apply to the underlying land cover maps used to produce land change estimates.
