# Supplementary material for: Tracking cropland transitions: A comparative analysis of U.S. land cover change data
Source: PLoS One. 2025 Mar 18;20(3):e0313880. doi: 10.1371/journal.pone.0313880 (PMC11918356; doi:10.1371/journal.pone.0313880)
Supplement: S2 Table — (DOCX) [file pone.0313880.s002.docx]

S2 Table. LCMAP land cover definitions.

| **LCMAP Level 1 Class** | **NLCD Level 2 Class** |
| --- | --- |
| Developed | Developed, Open Space  Developed, Low Intensity  Developed, Medium Intensity  Developed, High Intensity |
| Cropland | Pasture/Hay  Cultivated Crops |
| Grass/Shrub | Dwarf Scrub  Shrub/Scrub  Grassland/Herbaceous  Sedge/Herbaceous  Lichens  Moss |
| Tree Cover | Deciduous Forest  Evergreen Forest  Mixed Forest |
| Water | Open Water |
| Wetland | Woody Wetlands  Emergent Herbaceous Wetlands |
| Ice/Snow | Perennial Ice/Snow |
| Barren | Barren Land |
