# Supplementary material for: Tracking cropland transitions: A comparative analysis of U.S. land cover change data
Source: PLoS One. 2025 Mar 18;20(3):e0313880. doi: 10.1371/journal.pone.0313880 (PMC11918356; doi:10.1371/journal.pone.0313880)
Supplement: S3 Table — (DOCX) [file pone.0313880.s003.docx]

S3 Table. Expanded cropland class definitions across datasets.

| **Product** | **Included in cropland definition** | **Excluded from cropland definition** | **Source** |
| --- | --- | --- | --- |
| **LCMAP** | - Cultivated and uncultivated land used for fiber, food, and fuels - Hay and managed pasture lands (corresponding to NLCD Pasture/Hay class) - Perennial woody crops including orchards and vineyards - Confined livestock operations | - Land predominately covered with shrubs or grasses (rangeland) | Defined in product guide with correspondence to NLCD Level 2 classes |
| **Potapov *et al.*** | - Cultivated and uncultivated land used for fiber, food, and fuels - Forage, including hay - Cultivated fallow land (up to four years) | - Permanent managed pasture - Perennial woody crops including orchards and vineyards - Shifting cultivation | Described as “largely consistent with the arable land category reported by the Food and Agriculture Organization (FAO)” |
| **Lark *et al.*** | - Cultivated and uncultivated land used for fiber, food, and fuels - Cultivated fallow land - Alfalfa - Perennial woody crops including orchards and vineyards | - Hay and managed pasture lands (corresponding to NLCD Pasture/Hay class) | Defined according to underlying CDL definitions |
| **NRI** | - Cultivated and uncultivated land used for fiber, food, and fuels - Permanent hayland - Pastureland in rotation - Perennial woody crops including orchards and vineyards | - CRP land - Permanent pasture - Rangeland |  |
