# Supplementary material for: Tracking cropland transitions: A comparative analysis of U.S. land cover change data
Source: PLoS One. 2025 Mar 18;20(3):e0313880. doi: 10.1371/journal.pone.0313880 (PMC11918356; doi:10.1371/journal.pone.0313880)
Supplement: S4 Table — (DOCX) [file pone.0313880.s004.docx]

S4 Table. Pixelwise long-term pattern class agreement between LCMAP and Lark et al. 2020 in thousands of acres from 2008 - 2017.

|  | LCMAP (thousand acres) | | | | | | |
| --- | --- | --- | --- | --- | --- | --- | --- |
| Lark *et al.* 2020 |  | **Cropland abandonment** | **Cropland expansion** | **Intermittent cropland** | **Stable cropland** | **Stable non-cropland** | **Total** |
|  | **Cropland abandonment** | 146.5 | 21.2 | 0.4 | 2345.4 | 181.3 | **2694.8** |
|  | **Cropland expansion** | 34.0 | 1823.1 | 0.2 | 5177.8 | 1414.2 | **8449.3** |
|  | **Intermittent cropland** | 295.7 | 747.3 | 0.5 | 13672.4 | 3267.5 | **17983.4** |
|  | **Stable cropland** | 673.5 | 1244.3 | 1.3 | 280690.3 | 5863.6 | **288473.1** |
|  | **Stable non-cropland** | 2001.7 | 4499.5 | 2.5 | 160850.3 | 1439719.1 | **1607073.1** |
|  | **Total** | **3151.5** | **8335.4** | **4.8** | **462736.2** | **1450445.7** | **1924673.6** |
