# Supplementary material for: Tracking cropland transitions: A comparative analysis of U.S. land cover change data
Source: PLoS One. 2025 Mar 18;20(3):e0313880. doi: 10.1371/journal.pone.0313880 (PMC11918356; doi:10.1371/journal.pone.0313880)
Supplement: S5 Table — (DOCX) [file pone.0313880.s005.docx]

S5 Table. Kappa coefficient determination of agreement between LCMAP and Lark et al. 2020.

|  | Probability of chance agreement | Observed agreement | Kappa coefficient (κ) |
| --- | --- | --- | --- |
| Cropland abandonment | 0.000002 | 0.000076 | - |
| Cropland expansion | 0.000019 | 0.000947 | - |
| Intermittent cropland | 0.000000 | 0.000000 | - |
| Stable cropland | 0.036035 | 0.145838 | - |
| Stable non-cropland | 0.629250 | 0.748033 | - |
| Total | 0.665306 | 0.894894 | **0.6859649** |
