# Supplementary material for: Tracking cropland transitions: A comparative analysis of U.S. land cover change data
Source: PLoS One. 2025 Mar 18;20(3):e0313880. doi: 10.1371/journal.pone.0313880 (PMC11918356; doi:10.1371/journal.pone.0313880)
Supplement: S6 Table — For the cropland expansion and cropland abandonment disagreement, percentages indicate the fraction of all pixels in the entire dataset. For this reason, we do not calculate overall agreement or disagreement attributable to cropland expansion and cropland abandonment. Overall agreement indicates the extent to which pixelwise values between these datasets agree; overall disagreement indicates the extent to which pixelwise values disagree. Quantity and allocation disagreement together make up the overall disagreement, with quantity disagreement indicating the portion of the overall disagreement attributable to differences in the number of pixels each dataset assigns to each class. (DOCX) [file pone.0313880.s006.docx]

S6 Table. Agreement measures for comparison between LCMAP and both Lark et al. 2020.

|  | Total agreement | Total disagreement | Quantity disagreement | Allocation disagreement |
| --- | --- | --- | --- | --- |
| Cropland abandonment | - | - | 0.02% | 0.26% |
| Cropland expansion | - | - | 0.01% | 0.68% |
| Intermittent cropland | - | - | 0.93% | 0.00% |
| Stable cropland | - | - | 9.05% | 0.81% |
| Stable non-cropland | - | - | 8.14% | 1.11% |
| Total | 89.49% | 10.51% | 9.08% | 1.43% |

For the cropland expansion and cropland abandonment disagreement, percentages indicate the fraction of all pixels in the entire dataset. For this reason, we do not calculate overall agreement or disagreement attributable to cropland expansion and cropland abandonment. Overall agreement indicates the extent to which pixelwise values between these datasets agree; overall disagreement indicates the extent to which pixelwise values disagree. Quantity and allocation disagreement together make up the overall disagreement, with quantity disagreement indicating the portion of the overall disagreement attributable to differences in the number of pixels each dataset assigns to each class.
