# Supplementary figures and images for: Tracking cropland transitions: A comparative analysis of U.S. land cover change data
Source: PLoS One. 2025 Mar 18;20(3):e0313880. doi: 10.1371/journal.pone.0313880 (PMC11918356; doi:10.1371/journal.pone.0313880)

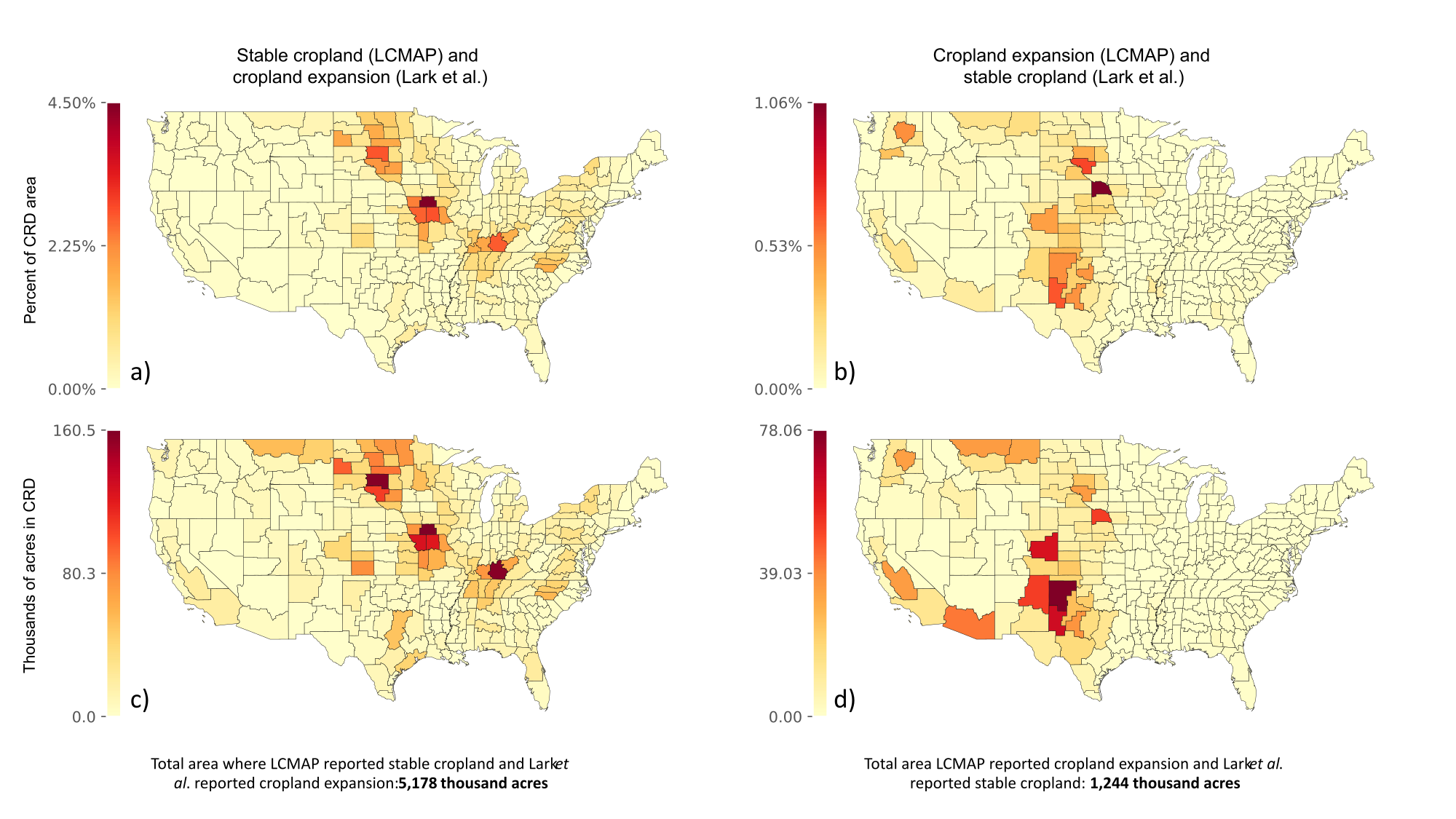

Supplement: S1 Fig — The first column of this figure shows the spatial distribution of pixels where LCMAP reported stable cropland and Lark et al. reported cropland expansion. The second column shows the reciprocal relationship, where LCMAP reported cropland expansion and Lark et al. 2020 reported stable cropland. Note that each subplot has a different scale and that the first row is in terms of the percent of the CRD area, while the second is in thousands of acres. (TIF) [file pone.0313880.s007.tif]

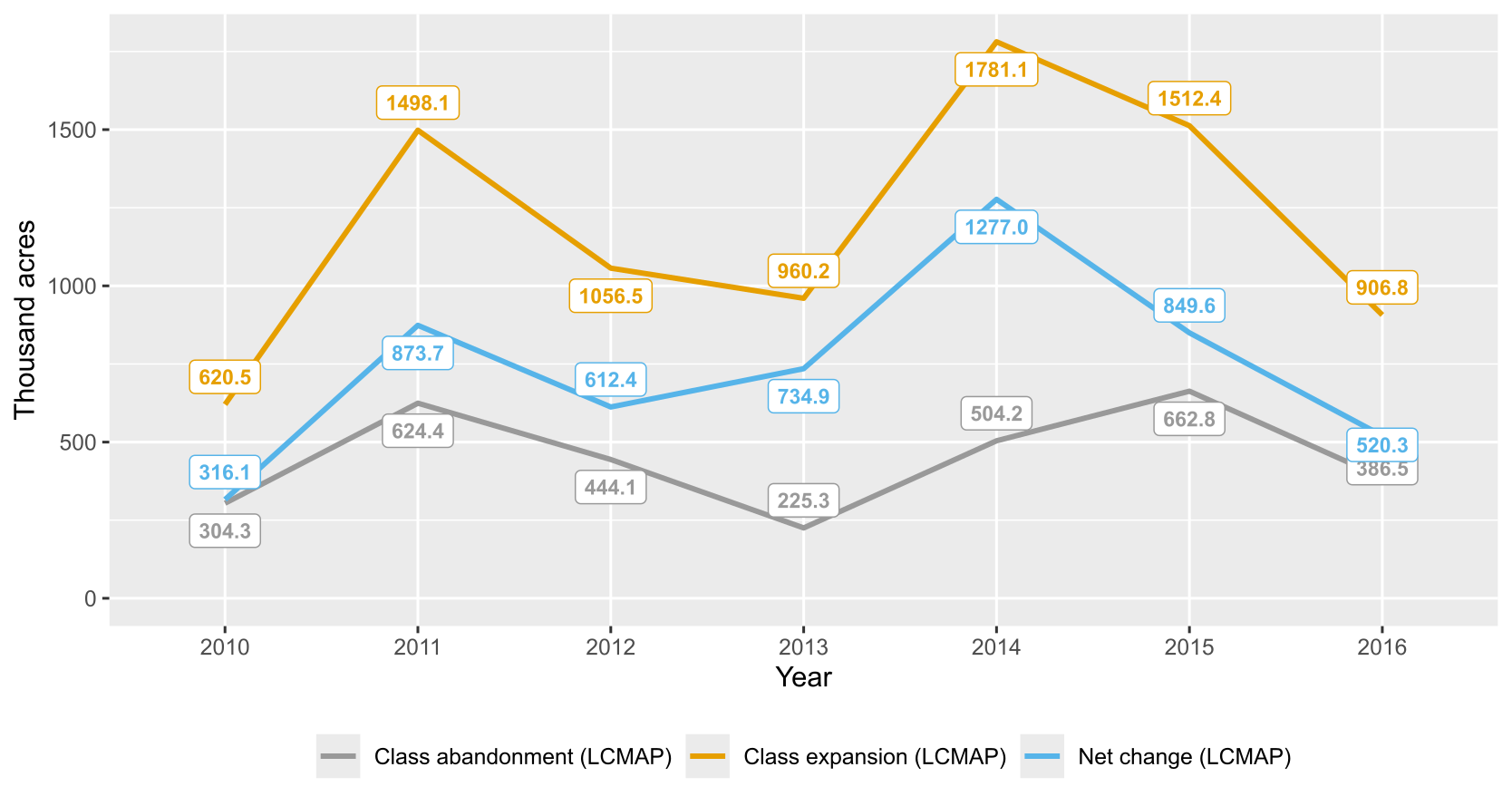

Supplement: S2 Fig — (TIF) [file pone.0313880.s008.tif]

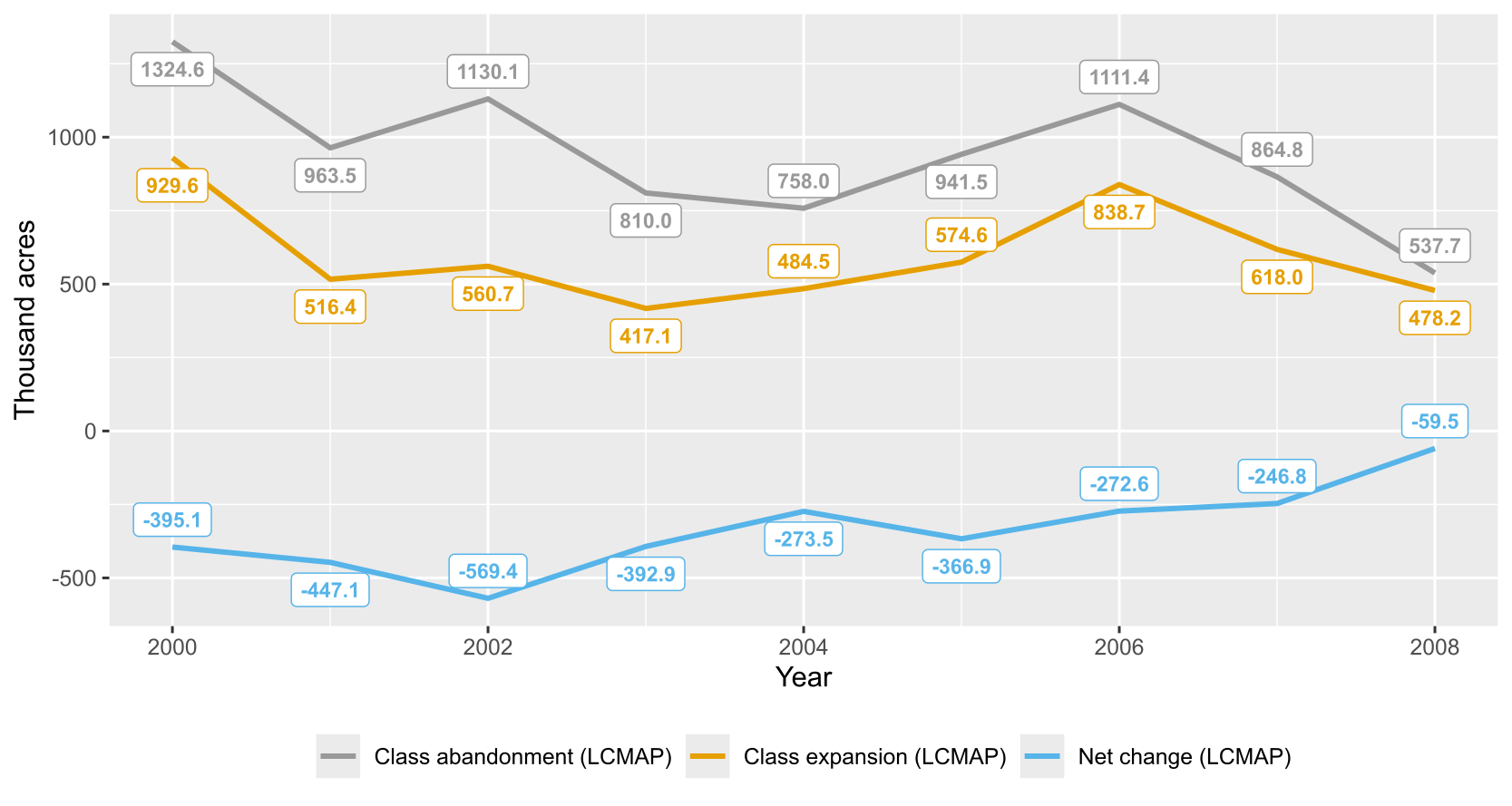

Supplement: S3 Fig — This figure shows the quantity (in thousands of acres) of cropland expansion and abandonment between 1998 and 2009. Because of how we define long-term pattern classes (Table 3), for the 1998 – 2009 interval, the first potential year of a class change is 2000 and the last is 2008. We performed this analysis separately from the main analysis described earlier in the paper using the same methods. (TIF) [file pone.0313880.s009.tif]
