# Supplementary material for: Tracking cropland transitions: A comparative analysis of U.S. land cover change data
Source: PLoS One. 2025 Mar 18;20(3):e0313880. doi: 10.1371/journal.pone.0313880 (PMC11918356; doi:10.1371/journal.pone.0313880)
Supplement: Attachment — Submitted filename: Response_to_Reviewers.docx [file pone.0313880.s010.docx]

Thank you for the opportunity to review the manuscript. We feel that the reviewer comments were largely supportive and constructive. We have worked to respond to all of the comments (blue text) and explained our rationale below. Thank you again, and we look forward to hearing from you.

PONE-D-24-19267

Tracking cropland transitions: a comparative analysis of U.S. land cover change data

PLOS ONE

Dear Dr. Martin,

Thank you for submitting your manuscript to PLOS ONE. After careful consideration, we feel that it has merit but does not fully meet PLOS ONE’s publication criteria as it currently stands. Therefore, we invite you to submit a revised version of the manuscript that addresses the points raised during the review process.

Two reviewers have evaluated your article and they both feel it still needs to be revised before it can be considered again for publication in PLOSONE.

The first reviewer had positive feedback on the quality and relevance of the paper, and recommended a minor revision incorporating a few errors he has highlighted on certain aspects of wording and language in various sections of the text. He also advices the authors to take into consideration the fact that various imagery data sources have inherently different spatial resolution levels, and that needs to be taken into account when making comparative inferences.

We have revised according to these suggestions and detail those below.

The second reviewer had more profound concerns about the technical soundness and organization structure as well as general presentation of the articles and, although did not recommend rejection, suggested much more in-depth modifications in almost all sections. In particular, the reviewer points out that the article appears to be primarily based on the Lack et al (2020) dataset yet the authors claim it to be founded on the Cropland Data Layer dataset. This needs to be clarified and the write-up, including all discussion and conclusion amended accordingly.

We detail our responses below. Our paper is not primarily based on Lark et al. (2020), it is primarily based on the LCMAP, which we compare with three datasets, one of which is indeed Lark et al. (2020), which is based on the CDL. We have revised the paper to make it clear that we’re comparing our results with Lark et al. (2020), among others, and not the raw CDL. Apologies for the confusion.

There is also need to account for the temporal aspects including overlaps (annual, semi-annual etc.)  as well as longevities of the datasets sources (LCMAP, Lark et al; NRI etc.) before appropriate comparisons may be made and discussed with accuracy.

We have re-written the methods to improve transparency and accuracy as this is precisely what we attempted to do but did not explain it adequately in the initial submission. Additionally, we ensured that the data in the figures and tables reflected a unified approach to temporal harmonization.

It must be adequately justified why the NRI and Potapov datasets were included in the article since they received much less treatment, or appear to be not as central to the comparisons and discussion as are the LCMAP and Lark et al components. Similarly, NAIP and NLCD datasets seem to be only of peripheral significance to the study.

This is clarified in the revisions. The Lark vs. LCMAP comparison appears to be central because the Lark estimates (2020) have been driving much of the debate and the discussion in the literature. So we felt it was appropriate to focus on that comparison. Additionally, given the similarities in both the underlying datasets (CDL and LCMAP) and the processing methodologies, we were able to perform a pixelwise comparison between Lark and LCMAP that we could not reasonably do for NRI and Potapov as well.

The NRI is generally considered the best overall estimate, but it is not a remotely sensed dataset and thus is not available at the pixel scale that the Lark et al. (2020) and LCMAP datasets are. Potapov is included because it is the most recent of the four estimates and because it has a high spatial resolution.

The NLCD is not included because it is not independent of the CDL (as explained in the original submission (page 5): “Furthermore, we omit NLCD because the CDL leverages NLCD for its non-crop classification and validation such that it would not be an independent comparison.”) NAIP is not included because it’s a repository of photographic images that have to be viewed one at a time (usually for validation purposes) and have not been stitched into a national or regional database to date to our knowledge. Thus, each has its reason for inclusion and omission, and we have fleshed that out more in the revisions by adding a section on dataset selection in the methods.

Material in Discussion and Results sections must not be allowed to overlap/spill over into each other.

We’ve revised the manuscript throughout to clean this up.

Be sure to add line numbers throughout the text pages.

We apologize for that omission; we have added page and line numbers.

Reproduce illustrations with more clearly distinct reference information (legends and captions) making sure to make figure more explicitly color-code separated, remembering that some readers are color-blind. Also avoid footnotes throughout.

We’ve cleaned up all the figures.

To reduce overall clutter and number of illustrations, try to merge and collapse closely related tables together, and try to consolidate figures presenting related information into separate panels of same figures if possible.

We have merged several tables and figures together and replaced complicated or confusing figures with tables. Additionally, we have moved several tables to the supplementary materials.

Follow the author guidelines on the formatting style of PLOSONE to the letter, including rules for formatting tables and figures as well as reference listing.

Please submit your revised manuscript by Sep 21 2024 11:59PM. If you will need more time than this to complete your revisions, please reply to this message or contact the journal office at [plosone@plos.org](mailto:plosone@plos.org). When you're ready to submit your revision, log on to [https://www.editorialmanager.com/pone/](https://gcc02.safelinks.protection.outlook.com/?url=https%3A%2F%2Fwww.editorialmanager.com%2Fpone%2F&data=05%7C02%7CClark.Christopher%40epa.gov%7C52dd1275dfd546909cd408dcb8ae08cd%7C88b378b367484867acf976aacbeca6a7%7C0%7C0%7C638588305692467213%7CUnknown%7CTWFpbGZsb3d8eyJWIjoiMC4wLjAwMDAiLCJQIjoiV2luMzIiLCJBTiI6Ik1haWwiLCJXVCI6Mn0%3D%7C0%7C%7C%7C&sdata=W9VBxfng5mo4xBTdx6G%2Bem9UHrXkv5L6cBosZY3dXAY%3D&reserved=0) and select the 'Submissions Needing Revision' folder to locate your manuscript file.

Please include the following items when submitting your revised manuscript:

- A rebuttal letter that responds to each point raised by the academic editor and reviewer(s). You should upload this letter as a separate file labeled 'Response to Reviewers'.
- A marked-up copy of your manuscript that highlights changes made to the original version. You should upload this as a separate file labeled 'Revised Manuscript with Track Changes'.
- An unmarked version of your revised paper without tracked changes. You should upload this as a separate file labeled 'Manuscript'.

If you would like to make changes to your financial disclosure, please include your updated statement in your cover letter. Guidelines for resubmitting your figure files are available below the reviewer comments at the end of this letter.

If applicable, we recommend that you deposit your laboratory protocols in protocols.io to enhance the reproducibility of your results. Protocols.io assigns your protocol its own identifier (DOI) so that it can be cited independently in the future. For instructions see: [https://journals.plos.org/plosone/s/submission-guidelines#loc-laboratory-protocols](https://gcc02.safelinks.protection.outlook.com/?url=https%3A%2F%2Fjournals.plos.org%2Fplosone%2Fs%2Fsubmission-guidelines%23loc-laboratory-protocols&data=05%7C02%7CClark.Christopher%40epa.gov%7C52dd1275dfd546909cd408dcb8ae08cd%7C88b378b367484867acf976aacbeca6a7%7C0%7C0%7C638588305692476683%7CUnknown%7CTWFpbGZsb3d8eyJWIjoiMC4wLjAwMDAiLCJQIjoiV2luMzIiLCJBTiI6Ik1haWwiLCJXVCI6Mn0%3D%7C0%7C%7C%7C&sdata=dPpzoOurYHPe0ejmQmqloicItteRdAvrSA43XGnvpXk%3D&reserved=0). Additionally, PLOS ONE offers an option for publishing peer-reviewed Lab Protocol articles, which describe protocols hosted on protocols.io. Read more information on sharing protocols at [https://plos.org/protocols?utm_medium=editorial-email&utm_source=authorletters&utm_campaign=protocols](https://gcc02.safelinks.protection.outlook.com/?url=https%3A%2F%2Fplos.org%2Fprotocols%3Futm_medium%3Deditorial-email%26utm_source%3Dauthorletters%26utm_campaign%3Dprotocols&data=05%7C02%7CClark.Christopher%40epa.gov%7C52dd1275dfd546909cd408dcb8ae08cd%7C88b378b367484867acf976aacbeca6a7%7C0%7C0%7C638588305692484260%7CUnknown%7CTWFpbGZsb3d8eyJWIjoiMC4wLjAwMDAiLCJQIjoiV2luMzIiLCJBTiI6Ik1haWwiLCJXVCI6Mn0%3D%7C0%7C%7C%7C&sdata=p3doZQbDQ80dqguklJuf%2FpLRYzAag59F8lpvPLQq3J4%3D&reserved=0).

We look forward to receiving your revised manuscript.

Kind regards,

Nickson E. Otieno
Academic Editor
PLOS ONE

Journal requirements:

When submitting your revision, we need you to address these additional requirements.

1. Please ensure that your manuscript meets PLOS ONE's style requirements, including those for file naming. The PLOS ONE style templates can be found at
[https://journals.plos.org/plosone/s/file?id=wjVg/PLOSOne_formatting_sample_main_body.pdf](https://gcc02.safelinks.protection.outlook.com/?url=https%3A%2F%2Fjournals.plos.org%2Fplosone%2Fs%2Ffile%3Fid%3DwjVg%2FPLOSOne_formatting_sample_main_body.pdf&data=05%7C02%7CClark.Christopher%40epa.gov%7C52dd1275dfd546909cd408dcb8ae08cd%7C88b378b367484867acf976aacbeca6a7%7C0%7C0%7C638588305692491084%7CUnknown%7CTWFpbGZsb3d8eyJWIjoiMC4wLjAwMDAiLCJQIjoiV2luMzIiLCJBTiI6Ik1haWwiLCJXVCI6Mn0%3D%7C0%7C%7C%7C&sdata=gDVl%2BwPZ23jTYexX1oOUoO2jarAAzfVC6njow47S%2F9U%3D&reserved=0) and
[https://journals.plos.org/plosone/s/file?id=ba62/PLOSOne_formatting_sample_title_authors_affiliations.pdf](https://gcc02.safelinks.protection.outlook.com/?url=https%3A%2F%2Fjournals.plos.org%2Fplosone%2Fs%2Ffile%3Fid%3Dba62%2FPLOSOne_formatting_sample_title_authors_affiliations.pdf&data=05%7C02%7CClark.Christopher%40epa.gov%7C52dd1275dfd546909cd408dcb8ae08cd%7C88b378b367484867acf976aacbeca6a7%7C0%7C0%7C638588305692497742%7CUnknown%7CTWFpbGZsb3d8eyJWIjoiMC4wLjAwMDAiLCJQIjoiV2luMzIiLCJBTiI6Ik1haWwiLCJXVCI6Mn0%3D%7C0%7C%7C%7C&sdata=V7TxOJ9jgWgjl%2BRCIuzO20dhCDZypXX1rGId61wHUHE%3D&reserved=0)

2. Thank you for stating the following financial disclosure: 
 [RTI received financial support for this work from EPA’s Office of Research and Development (<https://www.epa.gov/aboutepa/about-office-research-and-development-ord>) under contract 68HERD20A0004/68HERH22F0010. The views presented are those of the authors and do not necessarily represent the views or policies of the US Environmental Protection Agency.].  
Please state what role the funders took in the study.  If the funders had no role, please state: "The funders had no role in study design, data collection and analysis, decision to publish, or preparation of the manuscript." 
If this statement is not correct you must amend it as needed. 
Please include this amended Role of Funder statement in your cover letter; we will change the online submission form on your behalf.

We’ve added the following: “The funders participated in the study design, interpretation of results, and in manuscript preparation.”

3. We note that Figures 7 and 9 in your submission contain [map/satellite] images which may be copyrighted. All PLOS content is published under the Creative Commons Attribution License (CC BY 4.0), which means that the manuscript, images, and Supporting Information files will be freely available online, and any third party is permitted to access, download, copy, distribute, and use these materials in any way, even commercially, with proper attribution. For these reasons, we cannot publish previously copyrighted maps or satellite images created using proprietary data, such as Google software (Google Maps, Street View, and Earth). For more information, see our copyright guidelines: [http://journals.plos.org/plosone/s/licenses-and-copyright](https://gcc02.safelinks.protection.outlook.com/?url=http%3A%2F%2Fjournals.plos.org%2Fplosone%2Fs%2Flicenses-and-copyright&data=05%7C02%7CClark.Christopher%40epa.gov%7C52dd1275dfd546909cd408dcb8ae08cd%7C88b378b367484867acf976aacbeca6a7%7C0%7C0%7C638588305692504549%7CUnknown%7CTWFpbGZsb3d8eyJWIjoiMC4wLjAwMDAiLCJQIjoiV2luMzIiLCJBTiI6Ik1haWwiLCJXVCI6Mn0%3D%7C0%7C%7C%7C&sdata=CUk4Gs%2F4hQPGCvkHIp4ADA0EKXCXd3jTfPTiADFIqJc%3D&reserved=0).
 
We require you to either (1) present written permission from the copyright holder to publish these figures specifically under the CC BY 4.0 license, or (2) remove the figures from your submission:

Figures 7 and 9 are original work from this study and do not require copyright by anyone external.

1. You may seek permission from the original copyright holder of Figures 7 and 9  to publish the content specifically under the CC BY 4.0 license.  
 
We recommend that you contact the original copyright holder with the Content Permission Form ([http://journals.plos.org/plosone/s/file?id=7c09/content-permission-form.pdf](https://gcc02.safelinks.protection.outlook.com/?url=http%3A%2F%2Fjournals.plos.org%2Fplosone%2Fs%2Ffile%3Fid%3D7c09%2Fcontent-permission-form.pdf&data=05%7C02%7CClark.Christopher%40epa.gov%7C52dd1275dfd546909cd408dcb8ae08cd%7C88b378b367484867acf976aacbeca6a7%7C0%7C0%7C638588305692511108%7CUnknown%7CTWFpbGZsb3d8eyJWIjoiMC4wLjAwMDAiLCJQIjoiV2luMzIiLCJBTiI6Ik1haWwiLCJXVCI6Mn0%3D%7C0%7C%7C%7C&sdata=8SJ57UlGR1Aknicj3zYmzTzEMdxI5mVNZJbmpMrgjoc%3D&reserved=0)) and the following text:
“I request permission for the open-access journal PLOS ONE to publish XXX under the Creative Commons Attribution License (CCAL) CC BY 4.0 ([http://creativecommons.org/licenses/by/4.0/](https://gcc02.safelinks.protection.outlook.com/?url=http%3A%2F%2Fcreativecommons.org%2Flicenses%2Fby%2F4.0%2F&data=05%7C02%7CClark.Christopher%40epa.gov%7C52dd1275dfd546909cd408dcb8ae08cd%7C88b378b367484867acf976aacbeca6a7%7C0%7C0%7C638588305692517762%7CUnknown%7CTWFpbGZsb3d8eyJWIjoiMC4wLjAwMDAiLCJQIjoiV2luMzIiLCJBTiI6Ik1haWwiLCJXVCI6Mn0%3D%7C0%7C%7C%7C&sdata=JhXlGKHKk776xwO72P24jQuBR4WLLgrVSGbtdwGUIF4%3D&reserved=0)). Please be aware that this license allows unrestricted use and distribution, even commercially, by third parties. Please reply and provide explicit written permission to publish XXX under a CC BY license and complete the attached form.”
 
Please upload the completed Content Permission Form or other proof of granted permissions as an ""Other"" file with your submission.
 
In the figure caption of the copyrighted figure, please include the following text: “Reprinted from [ref] under a CC BY license, with permission from [name of publisher], original copyright [original copyright year].”
 
2. If you are unable to obtain permission from the original copyright holder to publish these figures under the CC BY 4.0 license or if the copyright holder’s requirements are incompatible with the CC BY 4.0 license, please either i) remove the figure or ii) supply a replacement figure that complies with the CC BY 4.0 license. Please check copyright information on all replacement figures and update the figure caption with source information. If applicable, please specify in the figure caption text when a figure is similar but not identical to the original image and is therefore for illustrative purposes only.
The following resources for replacing copyrighted map figures may be helpful:
 
USGS National Map Viewer (public domain): [http://viewer.nationalmap.gov/viewer/](https://gcc02.safelinks.protection.outlook.com/?url=http%3A%2F%2Fviewer.nationalmap.gov%2Fviewer%2F&data=05%7C02%7CClark.Christopher%40epa.gov%7C52dd1275dfd546909cd408dcb8ae08cd%7C88b378b367484867acf976aacbeca6a7%7C0%7C0%7C638588305692524297%7CUnknown%7CTWFpbGZsb3d8eyJWIjoiMC4wLjAwMDAiLCJQIjoiV2luMzIiLCJBTiI6Ik1haWwiLCJXVCI6Mn0%3D%7C0%7C%7C%7C&sdata=9KUuU4X9J6XFV%2FtgqaeNKGQ0oZjARle8mSEKKrDFWAY%3D&reserved=0)
The Gateway to Astronaut Photography of Earth (public domain): [http://eol.jsc.nasa.gov/sseop/clickmap/](https://gcc02.safelinks.protection.outlook.com/?url=http%3A%2F%2Feol.jsc.nasa.gov%2Fsseop%2Fclickmap%2F&data=05%7C02%7CClark.Christopher%40epa.gov%7C52dd1275dfd546909cd408dcb8ae08cd%7C88b378b367484867acf976aacbeca6a7%7C0%7C0%7C638588305692530830%7CUnknown%7CTWFpbGZsb3d8eyJWIjoiMC4wLjAwMDAiLCJQIjoiV2luMzIiLCJBTiI6Ik1haWwiLCJXVCI6Mn0%3D%7C0%7C%7C%7C&sdata=cAdh%2BduAehHAs4lywd%2B17VuMc%2BaY4qf5nWluA71P2Lo%3D&reserved=0)
Maps at the CIA (public domain): [https://www.cia.gov/library/publications/the-world-factbook/index.html](https://gcc02.safelinks.protection.outlook.com/?url=https%3A%2F%2Fwww.cia.gov%2Flibrary%2Fpublications%2Fthe-world-factbook%2Findex.html&data=05%7C02%7CClark.Christopher%40epa.gov%7C52dd1275dfd546909cd408dcb8ae08cd%7C88b378b367484867acf976aacbeca6a7%7C0%7C0%7C638588305692537305%7CUnknown%7CTWFpbGZsb3d8eyJWIjoiMC4wLjAwMDAiLCJQIjoiV2luMzIiLCJBTiI6Ik1haWwiLCJXVCI6Mn0%3D%7C0%7C%7C%7C&sdata=iQovpknfaHmP%2F6c0w4UDc8TxrJI%2FGJ7p1c8KfCxzG1E%3D&reserved=0) and [https://www.cia.gov/library/publications/cia-maps-publications/index.html](https://gcc02.safelinks.protection.outlook.com/?url=https%3A%2F%2Fwww.cia.gov%2Flibrary%2Fpublications%2Fcia-maps-publications%2Findex.html&data=05%7C02%7CClark.Christopher%40epa.gov%7C52dd1275dfd546909cd408dcb8ae08cd%7C88b378b367484867acf976aacbeca6a7%7C0%7C0%7C638588305692543944%7CUnknown%7CTWFpbGZsb3d8eyJWIjoiMC4wLjAwMDAiLCJQIjoiV2luMzIiLCJBTiI6Ik1haWwiLCJXVCI6Mn0%3D%7C0%7C%7C%7C&sdata=8j3r1K0S7tIXXVNMGwTlckBPPa3%2B%2Fr8iCwsGI1P55rg%3D&reserved=0)
NASA Earth Observatory (public domain): [http://earthobservatory.nasa.gov/](https://gcc02.safelinks.protection.outlook.com/?url=http%3A%2F%2Fearthobservatory.nasa.gov%2F&data=05%7C02%7CClark.Christopher%40epa.gov%7C52dd1275dfd546909cd408dcb8ae08cd%7C88b378b367484867acf976aacbeca6a7%7C0%7C0%7C638588305692550445%7CUnknown%7CTWFpbGZsb3d8eyJWIjoiMC4wLjAwMDAiLCJQIjoiV2luMzIiLCJBTiI6Ik1haWwiLCJXVCI6Mn0%3D%7C0%7C%7C%7C&sdata=4bb7ozvKvYRCtJ7pEBCXqFB9eZQomKjXUOnO7kXaif4%3D&reserved=0)
Landsat: [http://landsat.visibleearth.nasa.gov/](https://gcc02.safelinks.protection.outlook.com/?url=http%3A%2F%2Flandsat.visibleearth.nasa.gov%2F&data=05%7C02%7CClark.Christopher%40epa.gov%7C52dd1275dfd546909cd408dcb8ae08cd%7C88b378b367484867acf976aacbeca6a7%7C0%7C0%7C638588305692557876%7CUnknown%7CTWFpbGZsb3d8eyJWIjoiMC4wLjAwMDAiLCJQIjoiV2luMzIiLCJBTiI6Ik1haWwiLCJXVCI6Mn0%3D%7C0%7C%7C%7C&sdata=NV59JniYslnIXpwl3YgGi%2BFn6tIHwgwCk8oRVrtOCG4%3D&reserved=0)
USGS EROS (Earth Resources Observatory and Science (EROS) Center) (public domain): [http://eros.usgs.gov/#](https://gcc02.safelinks.protection.outlook.com/?url=http%3A%2F%2Feros.usgs.gov%2F%23&data=05%7C02%7CClark.Christopher%40epa.gov%7C52dd1275dfd546909cd408dcb8ae08cd%7C88b378b367484867acf976aacbeca6a7%7C0%7C0%7C638588305692564401%7CUnknown%7CTWFpbGZsb3d8eyJWIjoiMC4wLjAwMDAiLCJQIjoiV2luMzIiLCJBTiI6Ik1haWwiLCJXVCI6Mn0%3D%7C0%7C%7C%7C&sdata=1Qbl8Rc6leYNgWvVaYDuR0CxTB%2Fjll7XUNLQ5xERGvQ%3D&reserved=0)
Natural Earth (public domain): [http://www.naturalearthdata.com/](https://gcc02.safelinks.protection.outlook.com/?url=http%3A%2F%2Fwww.naturalearthdata.com%2F&data=05%7C02%7CClark.Christopher%40epa.gov%7C52dd1275dfd546909cd408dcb8ae08cd%7C88b378b367484867acf976aacbeca6a7%7C0%7C0%7C638588305692570827%7CUnknown%7CTWFpbGZsb3d8eyJWIjoiMC4wLjAwMDAiLCJQIjoiV2luMzIiLCJBTiI6Ik1haWwiLCJXVCI6Mn0%3D%7C0%7C%7C%7C&sdata=Eii35AiPOvTCRMScIXec3vofgi1LJl1VFPYg6t3oJBY%3D&reserved=0)

4. Please review your reference list to ensure that it is complete and correct. If you have cited papers that have been retracted, please include the rationale for doing so in the manuscript text, or remove these references and replace them with relevant current references. Any changes to the reference list should be mentioned in the rebuttal letter that accompanies your revised manuscript. If you need to cite a retracted article, indicate the article’s retracted status in the References list and also include a citation and full reference for the retraction notice.

Additional Editor Comments (if provided):


[Note: HTML markup is below. Please do not edit.]

Reviewers' comments:

Reviewer's Responses to Questions

**Comments to the Author**

1. Is the manuscript technically sound, and do the data support the conclusions?

The manuscript must describe a technically sound piece of scientific research with data that supports the conclusions. Experiments must have been conducted rigorously, with appropriate controls, replication, and sample sizes. The conclusions must be drawn appropriately based on the data presented.

Reviewer #1: Yes

Reviewer #2: No

2. Has the statistical analysis been performed appropriately and rigorously?

Reviewer #1: Yes

Reviewer #2: No

3. Have the authors made all data underlying the findings in their manuscript fully available?

The [PLOS Data policy](https://gcc02.safelinks.protection.outlook.com/?url=http%3A%2F%2Fwww.plosone.org%2Fstatic%2Fpolicies.action%23sharing&data=05%7C02%7CClark.Christopher%40epa.gov%7C52dd1275dfd546909cd408dcb8ae08cd%7C88b378b367484867acf976aacbeca6a7%7C0%7C0%7C638588305692577336%7CUnknown%7CTWFpbGZsb3d8eyJWIjoiMC4wLjAwMDAiLCJQIjoiV2luMzIiLCJBTiI6Ik1haWwiLCJXVCI6Mn0%3D%7C0%7C%7C%7C&sdata=EejTEG%2BA2l%2BVnol%2BZCld%2Ff24u7jycK4cwoaQZvQa5hw%3D&reserved=0) requires authors to make all data underlying the findings described in their manuscript fully available without restriction, with rare exception (please refer to the Data Availability Statement in the manuscript PDF file). The data should be provided as part of the manuscript or its supporting information, or deposited to a public repository. For example, in addition to summary statistics, the data points behind means, medians and variance measures should be available. If there are restrictions on publicly sharing data—e.g. participant privacy or use of data from a third party—those must be specified.

Reviewer #1: Yes

Reviewer #2: No

4. Is the manuscript presented in an intelligible fashion and written in standard English?

PLOS ONE does not copyedit accepted manuscripts, so the language in submitted articles must be clear, correct, and unambiguous. Any typographical or grammatical errors should be corrected at revision, so please note any specific errors here.

Reviewer #1: Yes

Reviewer #2: Yes

5. Review Comments to the Author

Please use the space provided to explain your answers to the questions above. You may also include additional comments for the author, including concerns about dual publication, research ethics, or publication ethics. (Please upload your review as an attachment if it exceeds 20,000 characters)

Reviewer #1: Section Page # Para # Line # Comment/suggestion/question
Abstract 1 2 1 “……. fuel put pressure on ……..” instead of “….fuel influence the ……”

Suggested change made.

Introduction 3 3 2 Check that “frequency of data collection” and “time intervals of representation” are not used in the same context?

Fixed.

4 3 “…….reasons for these …….”

Suggested change made.

4 Table 1 For Global Cropland Expansion in the 21st Century (Potapov), the data is Landsat GLAD ARD and not MODIS NPP? Check [https://nature.com/articles/s43016-021-00429-z](https://gcc02.safelinks.protection.outlook.com/?url=https%3A%2F%2Fnature.com%2Farticles%2Fs43016-021-00429-z&data=05%7C02%7CClark.Christopher%40epa.gov%7C52dd1275dfd546909cd408dcb8ae08cd%7C88b378b367484867acf976aacbeca6a7%7C0%7C0%7C638588305692583984%7CUnknown%7CTWFpbGZsb3d8eyJWIjoiMC4wLjAwMDAiLCJQIjoiV2luMzIiLCJBTiI6Ik1haWwiLCJXVCI6Mn0%3D%7C0%7C%7C%7C&sdata=hyYo2c4hyZ5RDJcMWogsZptSp%2FJsqTBAETpIJzSMmlc%3D&reserved=0) and read the section “Methods”. Also check the interval, which is four year and not semi annual?

Added Landsat ARD to data source in table.

5 1 9 …….to understand the ………..

Suggested change made.

2 1 “………for the conterminous…”

Suggested change made.

2. Methods 7 1 1 ‘…….product.for the CONUS based on time-series…….”

Suggested change made.

10 1 What proportion this would be of the total cropland area? Check results for this.

We did not include an assessment of the impact of the MMU on the total cropland area, as this paper seeks primarily to follow the recommendations from Lark *et al*. 2017 and Lark *et al*. 2020.

Figure 2 It is confusing and hard to read and understand. May be re-structured for better understanding.

Figure 2 replaced with table.

18 1 4 What about conversion to other land covers such as built up area and shrublands?

All conversions are from a non-cropland class to cropland or from cropland to a non-cropland class. Beyond that level of detail, we did not distinguish between specific conversions.

Table 6 Include area unit (000 acres) in column one. E.g. Cropland abandonment (000 acres)

Suggested change made.

In Potapov et al., Will adding managed pastures to the stable croplands from non cropland, be not closer to the LCMAP estimates? Check that. This will mean a definition variation/error.

Added table to clarify definitions of cropland across datasets.

27 1 6 “…….extent possible – definitions……. How?

Added table to detail harmonization of both definitions and temporal availability.

2 5 Check that managed grasslands are included in the croplands in Potapov et al.?

Some managed grassland—specifically managed hayland—is included in Potapov *et al*.

28 3 1 Quantities or “extent” in “………higher quantities of stable ….”

Suggested change made.

General comments The comparison is not at one spatial scale, as various imagery sources have different spatial resolution. How this spatial resolution in the range of 30m to 0.5 meter was addressed for uniformity?

Except for the NRI, all datasets are at 30m resolution. For spatial comparisons, we aggregated all the datasets to the resolution of the NRI (Cropland Reporting Districts, CRD) prior to comparing. For visual display purposes, all four datasets are displayed at the CRD-level. There are no datasets in the comparison that are available at a 0.5 meter resolution.

It would have been more appropriate to have used independent reference data for accuracy check of the various products, and then compared those among each other and with that of LCMAP. The review article projects that LCMAP is used as a reference. However, considering that LCMAP will have commission and omission errors, and will certainly needs an independent dataset to check with.

It is not the purpose of our study to independently validate all these datasets with our own data. Each of these datasets have already been validated by the original data-creators. The purpose of our study is to take the final validated estimates from these data sources, and put them through a common pre-processing (to harmonize to the degree possible spatial scales, temporal spans, and definitions) to compare their predictions of cropland expansion in the U.S. A “third party” independent validation of all these datasets with a common empirical dataset is an excellent idea, but one that would take years to undertake and it well outside the feasible scope for this manuscript. We are supportive of such an effort though, and in theory some subset of NAIP images (1000?) or private farm-level data from USDA could be used for such an undertaking.

Reviewer #2: The study assessed factors that drive changes in cropland estimation between Land Change Monitoring, Assessment, and Projection (LCMAP), National Resources Inventory (NRI), Cropland Data Layer (CDL, Lark et al. 2020), Potapov et al. (2022) datasets across coterminous United States. The study is interesting and important because of the various land cover datasets that exist and how differences between them could influence the outcome of their applications.

The study has several issues firstly with the “Cropland Data Layer”. The study does not use the Cropland Data Layer but the Lark et al 2020 dataset which is based on the Cropland Data Layer and National Land Cover Dataset. In some sections of the text it is mentioned as Cropland Data Layer e.g., the Abstract and Table 1 but in others as Lark et al 2020 dataset. It would be more appropriate to use just say dataset from Lark et al 2020 or something similar throughout the text just as the study states “dataset from Potapov et al. (2022)”.

We agree with the comment and have ensured that we refer to the dataset from Lark *et al*. 2020 (which is derived from the CDL) as Lark *et al*. for clarity; similarly, we refer to the dataset from Potapov *et al*. 2022 as Potapov *et al*.

The major issue is with the harmonisation or lack of with the cropland definitions and temporal scale of the datasets. Without the homogenisation of the cropland definitions and carrying out a sensitivity analysis it is not possible to say if the cropland estimation is based on the differences in definitions or some other factor. The temporal differences of the datasets raises the most concerns in the study. The LCMAP and Lark datasets are annual datasets and have a partial temporal overlap. The NRI dataset exists only for 2007, 2012, and 2017 and while 2007 is included in the analysis the LCMAP and Lark are from 2008 onwards. This could greatly influence the results or not since it's a 1 year difference but the study needs to account for this. The Potapov dataset used covers 2019 whereas the LCMAP, Lark, NRI datasets are till 2017. Again the study does not account for how this difference could/could not influence the results.

All these differences were harmonized to the degree possible, but that was not adequately explained in the initial submission. This has been completely re-written in more detail. Because each of the datasets are slightly different, the comparison with the LCMAP (our central interest) was done individually by dataset. We have clarified the focus of this study through the addition of a dataset selection section in the methods and by including a new table (Table 3) that focuses on harmonization between each compared dataset and the processed LCMAP data.

If the objective of the study is to compare different datasets then they would have to be harmonised to be comparable otherwise it raises inconsistencies and would not answer the aim of the study “The aim of this study is to understand how estimates of cropland change from LCMAP differ from estimates from other data products across a common time period (2008-2017) and which factors drive these differences.”

This assertion is not necessarily true. The objective of this study is to compare the estimates from the different datasets over a common temporal period. This is clarified in the revised introduction: “We do not harmonize the class definitions because not only would that require re-deriving the original datasets, but also because these differences are the focus of this study—we are interested in how definitional differences lead to different dynamics and patterns of estimated cropland expansion. This will enable future users of these datasets to understand their strengths and limitations and how they compare to alternatives to inform future research and analysis.”

It is not the objective of this study to actually harmonize the datasets. That would require an entirely different approach, including re-deriving the satellite information to a common set of definitions, and an independent third-party validation empirical dataset applied to all remotely-sensed datasets. It is our objective to compare these datasets precisely to illustrate side-by-side their differences so that the community of researchers, policymakers, and decision makers can move beyond the “this dataset says X and that dataset says Y.” None of these datasets are right or wrong, they are the outcome of a series of assumptions and decisions by teams of people. But they all use the same term “cropland.” Thus, it is precisely our objective to compare these (with some harmonization for differences in scale and temporal period), so that as a community we can understand what an increase in cropland means depending on what dataset is used, both in terms of the spatial location of where that change is occurring, and the type of change that is occurring. From the community of stakeholders we work with in the Federal Government, this is sorely needed in the literature.

The Results section reads more like a discussion while the Discussion reads like the results. These two sections need major improvements.

We have attempted to resolve the issues in the results and discussion sections.

Overall the study seems to be more of a comparison between LCMAP and Lark datasets with NRI and Potapov datasets being forced into the comparison.

Direct pixel-level comparison, as well as annual quantities of cropland change, between LCMAP and both NRI and Potapov are confounded by the temporal availability of these datasets. We have added an explanation of this in the methods.

The comments about specific issues are given below. Given the lack of line numbers, the page and paragraph numbers are used instead

Martin 1: “National Resources Inventory (NRI), (2) the Cropland Data Layer (CDL, Lark et al. 2020), and (3) a dataset from …” Here, CDL is within the () whereas everywhere else in the text the short forms are not included within the reference brackets e.g., Martin 3, paragraph 3: “the USGS National Land Cover Database (NLCD) (Homer 2020), the USDA Cropland Data Layer (CDL) (US Department of Agriculture 2021a) …”.

Suggested change made.

Martin 1: “LCMAP reports more stable cropland and less stable noncropland in all comparisons, likely due to its expansive definition of “cropland” including managed grasslands (pasture and hay),...” how can this be stated when the differences in the temporal scales of the datasets have not been controlled?

They have been, see above.

Martin 1: “We found that most of the pixel-level disagreements (86%) were due to definitional differences among datasets, whereas the remainder (14%) were from a variety of causes including spatial allocation.” this gives the impression that the comparison is between all the four datasets when that is clearly not the case.

Clarified that the pixel-level disagreements only apply to the comparison between LCMAP and Lark *et al*. given that we did not perform a pixel-level comparison with the other two datasets.

Martin 1: “This study also includes a brief assessment of land use change from 2000-2008, an often-overlooked period, and find that many of the studies focused on 2008 and after may omit large amounts of cropland expansion and abandonment.” I fail to see the point of this assessment, it would be better to focus on the main findings in the abstract.

We removed the line from the abstract. We added a short paragraph in the Discussion instead as this is not a central focus of the paper, but it is an important implication, that land use change did not begin in 2008.

Martin 3: paragraph 3 “These tools vary in their…” should it not be datasets instead of tools since datasets is generally used across the text?

Suggested change made.

Martin 3: paragraph 3 “USDA NASS annual surveys reported an increase in principal crop planted
area of 0.38 million hectares (US Department of Agriculture 2021b), while a study based on the CDL data reported a value of net cropland expansion nearly four times larger” is “principal crop planted
area” defined the same as “net cropland expansion”? Or do they mean different things and that's why there is a difference.

Line removed from introduction as it was unnecessary.

Martin 3: paragraph 3 “several of these datasets (e.g., NRI, CDL, Census, etc.) yielded similar results in” is etc. a dataset? what is the Census dataset?

The Census is the USDA Agricultural Census, which is probably the most well-known dataset for agricultural purposes. That said, we should not assume familiarity, and the sentence was out of place anyway upon further review. We have deleted this sentence and moved it to a paragraph in the Discussion, clarifying what the Census is.

Martin 4: Table 1 - The inclusion of National Agricultural Imagery Program (NAIP) and National Land Cover Database (NLCD) seems unnecessary. There appears to be footnotes below the table which are not permitted - [https://journals.plos.org/plosone/s/submission-guidelines](https://gcc02.safelinks.protection.outlook.com/?url=https%3A%2F%2Fjournals.plos.org%2Fplosone%2Fs%2Fsubmission-guidelines&data=05%7C02%7CClark.Christopher%40epa.gov%7C52dd1275dfd546909cd408dcb8ae08cd%7C88b378b367484867acf976aacbeca6a7%7C0%7C0%7C638588305692590916%7CUnknown%7CTWFpbGZsb3d8eyJWIjoiMC4wLjAwMDAiLCJQIjoiV2luMzIiLCJBTiI6Ik1haWwiLCJXVCI6Mn0%3D%7C0%7C%7C%7C&sdata=olzPLRMiq1dthAGanucEG74uUPftiAl7xDxYptaa1U0%3D&reserved=0). Additionally it might be more appropriate to move this to the “a. Data description” section.

NAIP and NLCD removed from table. Table footnotes removed throughout.

Martin 4: Table 1 - While Table 1 “Availability” states that Potapov dataset is “Semiannual (2000-2019)” the website [https://glad.umd.edu/dataset/croplands](https://gcc02.safelinks.protection.outlook.com/?url=https%3A%2F%2Fglad.umd.edu%2Fdataset%2Fcroplands&data=05%7C02%7CClark.Christopher%40epa.gov%7C52dd1275dfd546909cd408dcb8ae08cd%7C88b378b367484867acf976aacbeca6a7%7C0%7C0%7C638588305692598151%7CUnknown%7CTWFpbGZsb3d8eyJWIjoiMC4wLjAwMDAiLCJQIjoiV2luMzIiLCJBTiI6Ik1haWwiLCJXVCI6Mn0%3D%7C0%7C%7C%7C&sdata=5LDur2LUKmgiPDggI3eVcJYctk20%2FtjFpwN9vlNkr5Y%3D&reserved=0) indicates the the dataset is available at “four-year intervals (2000-2003, 2004-2007, 2008-2011, 2012-2015, and 2016-2019)”. Is the dataset available at semi annual and four year intervals?

Apologies for the confusion. We have made this much more explicit in the revisions.

Martin 5: paragraph 2 “In this study we compare LCMAP-derived … not be an independent comparison.” this entire paragraph reads as what was done in the study and would be better to include it in “the aim of the study” paragraph.

We moved this to the new section on “Dataset selection and key attributes” in the Methods.

Martin 5: paragraph 3 “Stehman et al. (2021) provides a robust assessment … while in the NLCD they were 65% and 67%” this section should be its own separate section and before the “the aim of the study” paragraph. Additionally, are there no other studies that compared land cover between different datasets? does not have to be limited to the US datasets/studies.

Most studies, to our knowledge, use one dataset to address a particular research question (e.g., how much land use change has contributed to erosion). In the release of the NLCD, they compare their estimates with a few others. The NRI does not do that, nor did Potapov. It is surprising that that isn’t more common. That said, there are a few that have (Reistma et al. 2016 [compared CDL with NASS], Dunn et al. 2017 [compared CDL with NAIP], Copenhaver et al. [compared NRI, USDA Census, and CDL]). We’ve added a short paragraph on those.

Martin 7: “a. Data description” the heading is data description but only the LCMAP data is described. What about the other datasets?

Section renamed to LCMAP description. Additional description of other datasets added in methods.

Martin 7: paragraph 1 “Although LCMAP has only been available since 2020, this dataset has found use across a range of applications (Healey and Rover 2022) (Xian et al. 2021) (Tollerud, Brown, and Loveland 2020).” All the references should be within one bracket ().

Fixed.

Martin 8: Table 2 - it would be better to keep this in the supplementary.

Moved to supplementary.

Martin 9 and 10: Table 3 and Figure 1 - Figure 1 is a good visual explanation. The contents of Table 3 can be included in Figure 1 to make things more concise since the two explain the same things.

Table 3 merged into Figure 1.

Martin 10: Again, footnotes are not permitted.

Fixed.

Martin 10: “c. Definitional differences” it would make more sense to have this section after “a. Data description”. This way cropland and non-cropland are described before talking about the long-term pattern class definitions.

Suggested change made.

Martin 11: paragraph 1 “(“National Resources Inventory Glossary | NRCS” n.d.)” this seems like an odd reference style.

Fixed.

Martin 11: Table 4 - Is there a reason “permanent managed pasture” is not included in cropland in the Potapov dataset when this subclass is included in cropland in LCMAP dataset? Or why “perennial woody crops including orchards and vineyards” is not included in cropland in the Potapov dataset when this subclass is included in cropland in Lark dataset? There’s mention of homogenisation in the discussion section Martin 27 paragraph 1 “To make a direct comparison we therefore harmonised—to the extent possible …” but there does not seem to be any homogenisation of the classes done. How would the results of the study change if the definitions are harmonised? Isn’t this what the study aims to do?

The purpose of this study is not to perfectly harmonize these datasets. The dataset from Potapov *et al*. 2022 was made with a set of definitional assumptions that we cannot change after the fact. We note definitional differences where they occur as they impact the estimated cropland change, but we are unable to change the class definitions used to create the dataset. It is not possible—or at least, it is certainly outside the scope of this study—to rederive the Potapov *et al*. cropland extent maps with a definition of cropland that includes perennial woody crops or excludes managed hay.

Martin 11 and 12: Table 4 and Figure 2 - Figure 2 is a good visual explanation. Again, the contents of Table 4 can be included in Figure 2 to make things more concise since the two explain the same things. Additionally, consider using hollow coloured boundaries to make things easier to read and add categories in the legend for the solid and dash black blocks.

Replaced Figure 2 and Table 4 with a more concise table of what is included/excluded to make it clearer.

Martin 12: “d. Aligning temporal differences” - How can the use of the 2007 NRI dataset be justified when it does not temporally overlap with the other datasets used? How can the use of the 2019 (2017-2019) Potapov dataset be justified when the other dataset used are just up to 2017? The abstract states “LCMAP reports more stable cropland and less stable noncropland in all comparisons, likely due to its expansive definition of “cropland” including managed grasslands (pasture and hay), though overall agreement remains high” this could also be due to the temporal scale of the datasets?

This is explained with the new section on “Aligning temporal differences with the LCMAP.”

Martin 16: Figure 4 - People who are colour-blind will have a hard time reading this. Consider using a different colour palette. Also, add the full form for “LTPC”.

All figures have been reproduced with the format required for PLOSOne and use colorblind-accessible colors.

Martin 16: paragraph 1 “compared with other sources, most evident in the comparison to Lark et al.
2020. We can attribute some … LCMAP estimates than estimates from Lark et al. 2020.” the Result section should state the results of this study, not compare it to the findings of other studies or why the results have a certain pattern, that's what the discussion section is for.

We’ve revised the entire paper to focus the results in the results section and discussion in the discussion section.

Also what would the estimates look like when the definitions are harmonised?

Presumably they would look more similar, since that was the main reason they differed. But such an undertaking is well outside the scope of this effort.

Martin 17: Table 6 - this provides an explanation of the results, it should be as a text in the discussion section.

We moved this to the Discussion but kept it as a table. We feel that it’s a nice way to summarize the information.

Martin 17-20: most of the text describes the meaning of the results which is not what this Result section is for.

See above, the paper has been revised per this and other similar comments.

Martin 18: Table 6 - Should be table 7 and should be the same style as the other tables.

Fixed.

Martin 21: Figure 5 - What is the point of this figure when the same is plotted in Figure 6?

We agree that it’s redundant for the main text. But we have moved to the supplemental since we feel it’s nice to have a graph with all the results for LCMAP in on place.

Martin 21: Again, footnotes are not permitted.

Fixed.

Martin 22: Figure 6 - captions are at the top of the figure. They should be at the bottom.

Fixed.

Martin 25: Figure 7 - People who are colour-blind will have a hard time reading this. The labels on the right are externally added and not aligned with images and are of higher resolution than other parts of the image.

All figures have been reproduced with the format required for PLOSOne and use colorblind-accessible colors.

Martin 26: Figure 8 - Should be figure 8. Again, people who are colour-blind will have a hard time reading this. The labels on the right are externally added and not aligned with images and are of higher resolution than other parts of the image.

All figures have been reproduced with the format required for PLOSOne and use colorblind-accessible colors.

Martin 27: The Discussion section from Martin 27 paragraph 1 to Martin 29 paragraph 1 is easy to follow and makes sense after that it seems to go on a tangent. Martin 29 paragraph 2 “The longer temporal record of … application in question.” why is this aspect about the temporal interval included here when the paragraph above talks about the temporal aspects.

Paragraph removed.

Martin 29: paragraph 3 “rules outlined by Lark et al. 2017 to classify pixels based on their temporal
trajectories into stable, expansion, abandonment, or intermittent classes. We note however that this is a
subjective approach, application- and dataset-specific, and one that could potentially have a significant
impact on the resulting output.” with this and the lack of harmonised definitions and time interval does the study even assess what factors are influencing the estimates?

We feel this has been clarified with the lengthy revisions. We are not harmonizing definitions; we are harmonizing the temporal period assessed and the spatial resolutions. We are interested in the different results that stem from the different definitions and feel that yields a deeper insight on the types and locations of land use changes occurring in the U.S.

Martin 34-35: Supplementary Figure 1 and 2 - People who are colour-blind will have a hard time reading this. The labels on the right are externally added and not aligned with images and of higher resolution than other parts of the image.

All figures have been reproduced with the format required for PLOSOne and use colorblind-accessible colors.

6. PLOS authors have the option to publish the peer review history of their article ([what does this mean?](https://gcc02.safelinks.protection.outlook.com/?url=https%3A%2F%2Fjournals.plos.org%2Fplosone%2Fs%2Feditorial-and-peer-review-process%23loc-peer-review-history&data=05%7C02%7CClark.Christopher%40epa.gov%7C52dd1275dfd546909cd408dcb8ae08cd%7C88b378b367484867acf976aacbeca6a7%7C0%7C0%7C638588305692607126%7CUnknown%7CTWFpbGZsb3d8eyJWIjoiMC4wLjAwMDAiLCJQIjoiV2luMzIiLCJBTiI6Ik1haWwiLCJXVCI6Mn0%3D%7C0%7C%7C%7C&sdata=JkQUzTkdDAiUF1zG0bOBGUoygiDLjEyUtEzZ%2FWaC518%3D&reserved=0)). If published, this will include your full peer review and any attached files.


If you choose “no”, your identity will remain anonymous but your review may still be made public.


**Do you want your identity to be public for this peer review?** For information about this choice, including consent withdrawal, please see our [Privacy Policy](https://gcc02.safelinks.protection.outlook.com/?url=https%3A%2F%2Fwww.plos.org%2Fprivacy-policy&data=05%7C02%7CClark.Christopher%40epa.gov%7C52dd1275dfd546909cd408dcb8ae08cd%7C88b378b367484867acf976aacbeca6a7%7C0%7C0%7C638588305692616459%7CUnknown%7CTWFpbGZsb3d8eyJWIjoiMC4wLjAwMDAiLCJQIjoiV2luMzIiLCJBTiI6Ik1haWwiLCJXVCI6Mn0%3D%7C0%7C%7C%7C&sdata=DtYGWDOHv9HPeWmffdnAdprVVWz7hufzXbyKLSkauLM%3D&reserved=0).

Reviewer #1: No

Reviewer #2: **Yes:** Vishesh L. Diengdoh
